# An Ion Discharge-Driven Thruster Based on a Lithium Niobate Piezoelectric Transformer

**DOI:** 10.3390/mi16030277

**Published:** 2025-02-27

**Authors:** Qiannan Tao, Xinshuai Wang, Yang Gu, Wei Li

**Affiliations:** 1 College of Integrated Circuit Science and Engineering, Nanjing University of Posts and Telecommunications, Nanjing 210023, China; qntao_lea@njupt.edu.cn (Q.T.); 1024223323@njupt.edu.cn (X.W.); yanggukevin@gmail.com (Y.G.); 2 Department of Mechanical Engineering, University of Vermont, Burlington, VT 05405, USA

**Keywords:** lithium niobate, transformer, discharge, ion wind, piezoelectric simulation

## Abstract

Microrobots, characterized by their small size, flexibility, and portability, have a diverse range of potential applications. However, microrobots’ actuation (piezoelectric ceramics, dielectric elastomers, ion winds, etc.) often requires a high voltage, typically hundreds of volts. The lithium niobate transformer (LNT), a piezoelectric voltage transformer, presents a promising solution for miniaturizing high-voltage power supplies due to its compact size, low weight, and high step-up ratio. This study explores the effects of structural parameters and external circuits on the resonant frequency and step-up ratio of the LNT through numerical simulations and experiments. The results indicate the following: (1) the second-order longitudinal vibration frequency of the lithium niobate (LN) plate inversely correlates with its length; (2) the thickness and width of the plate have minimal impact on the frequency; (3) the step-up ratio increases as the plate thickness decreases. The experimental results suggest that LN plates with a thickness of 1 mm are preferable due to the fragility of 0.5 mm plates, especially at the output end. Additionally, optimizing the input circuit enhances voltage amplification, allowing the LNT to generate sufficient output voltage for corona discharge. These findings highlight the potential of LNTs for efficiently and reliably powering small-scale devices.

## 1. Introduction

With the continuous progress of science and technology, the application of microrobots in medical, industrial, and service fields is widespread. Microrobots require a high degree of flexibility and precise control, making the design of their drive system crucial. At present, materials such as piezoelectric ceramics and dielectric elastomers are widely used in the drive systems of microrobots [1,2,3]. These materials induce deformation or displacement by an applied electric field to realize precise robot motion [4,5,6]. In addition, in recent years, ion wind generated by discharge has gained attention as a new type of propulsion [7]. Our flying microrobot achieves a record of 5.5 thrust-to-weight ratio, which opens possibilities for incorporating microelectronics, enabling autonomous flight functionality. The ion wind is the movement of the neutral particles propelled by energetic electrons induced by the gas discharge [8,9]. Macroscopically, the motion of particles is fluid flow [10]. The operating principle of these actuator materials typically requires high voltages, ranging from several hundred to several thousand volts [7,11,12].

The necessity for input voltages over several thousand volts introduces significant complexity to power systems. The complexity of high-voltage power supplies makes it difficult to assemble them in a miniaturized way [13]. One promising solution is to introduce a piezoelectric transformer (PT) [14,15]. Unlike conventional electromagnetic transformers, piezoelectric transformers offer advantages like small size, low weight, simple structure, high electromechanical power density, high transformation ratios, and no electromagnetic interference [16,17]. Piezoelectric materials possess a distinct electromechanical coupling mechanism that facilitates the efficient conversion of mechanical energy to electrical energy. Consequently, these materials are extensively employed in the development of a wide range of electromechanical devices, including sensors [18,19], resonators [20,21,22], and filters [23]. Piezoelectric materials can also be used for the piezoelectric nanogenerator [24]. Based on the piezoelectric and inverse piezoelectric effects, the piezoelectric materials are fabricated into step-up and step-down transformers. Piezo-ceramic transformers of the Rosen type are classic step-up transformers. Orthogonal polarization is performed on the input and output parts of the Rosen-type transformer. Piezoelectric ceramic transformers require polarization control following their fabrication [25], have limited power density, and are too weak to withstand ultrahigh voltages. Nakamura et al. [26] found that lithium niobate shows greater potential for making transformers. LN not only has low damping, low elasticity, and low electrical losses but also has higher electromechanical coupling coefficients than piezoelectric ceramics at specific orientations. There is no degradation observed regarding the *Q*-factor when working substantially.

For the application of LN in transformers, Nakamura et al. [26] demonstrated that the anisotropy of LN can be used to increase voltage. LN shows strong electromechanical coupling, but its anisotropic nature often causes mode coupling, complicating the design and requiring careful selection of crystal cuts to balance both. For LNTs, the rotary Y-cut is considered optimal, and it was demonstrated that the rotation angle at 128° is the best choice, where $k_{32}\times k_{33}$ is the maximum [15,26]. Benwell et al. [27] also used the 135° Y-cut LN as a step-up transformer. Nakamura et al. [26] confirmed that the maximum output voltage depends on the relationship between the surface on which its output electrodes are deposited and the polarity of the polar plate, proposing two preferred configurations that achieve voltage gains of up to 750.

When the transformer output voltage reaches thousands of volts, it ionizes the air and produces ion wind. Johnson et al. [15] successfully generated a gas discharge in atmospheric air using a LNT. This research demonstrates that LNTs can not only provide high voltage for ionization devices but also directly generate ion wind. Based on this finding, we propose that LNTs can serve not only as the power supply system for microrobots but can also be utilized to directly design propulsion systems powered by the ion wind they generate. The discharge modes used to generate ion wind include the corona discharge and dielectric barrier discharge (DBD) [28]. The corona discharge mostly occurs at the sharp emitter electrode with DC voltage and produces a localized and intense ion wind. The DBD is typically actuated by the AC voltage [29]. The electrodes are separated by the dielectric and produce a uniform and stable plasma stream [30]. The unique advantages of ion winds, such as their low noise, low power consumption, fast response time, and absence of mechanical moving parts, are the reason for their potential use across a wide range of applications. For instance, ion wind generated by atmospheric air discharge can serve as a thruster [31,32]. Although ion wind thrust efficiency needs improvement, the simple discharge structure allows it to drive microrobot movement [33].

Although LNTs show great potential for low-voltage drives and miniaturized high-voltage applications, there are still several important aspects that need to be explored and investigated in depth. This paper addresses key aspects of LNTs that remain underexplored, including a detailed analysis of structural and circuit parameters, which can guide practical design. In addition, based on previous microrobot designs in our group, we propose two concrete application solutions. We focus on optimizing LNT performance under low input voltage conditions, utilizing a 128° Y-cut LN plate and optimizing the external circuit to successfully achieve corona discharge. The finite element method is used to simulate and analyze various LNT sizes and circuits, offering both theoretical and practical insights for their use in high-voltage power supplies.

## 2. Experiments

### 2.1. Theory and Experimental Method

**Figure 1:**
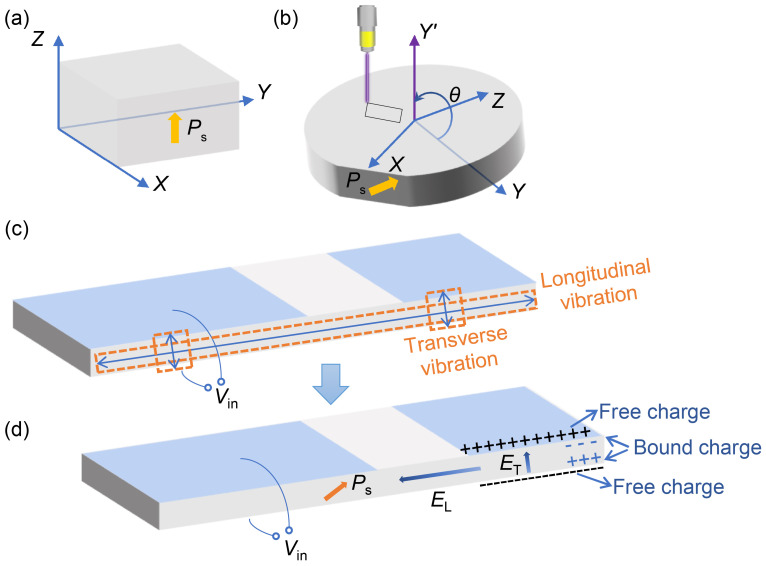
Illustration of the transformer principle for LNTs: (**a**) Crystal coordinate system of LiNbO_3_. (**b**) Rotated Y-cut LiNbO_3_ plate and the process of laser cutting. (**c**) Principle for the inverse piezoelectric effect of LNTs. (**d**) Principle for the positive piezoelectric effect of LNTs.

Unlike conventional anisotropic Rosen-type piezoelectric transformers, LNTs are not orthogonally polarized. The spontaneous polarization $P_{s}$ is parallel to the crystallographic *Z* axis in the single crystal of LiNbO_3_ [26], as shown in Figure 1a. The XYZ coordinates represent the crystal coordinate system. Thus, there is no output voltage generated if the input AC voltage is applied to the upper and lower surfaces of the LN plate of which the spontaneous polarization is along the direction of the thickness, according to the inverse/positive piezoelectric effect of piezoelectric materials. However, in a LiNbO_3_ plate with an orientation as shown in Figure 1b, the mechanical vibration caused by the inverse piezoelectric effect will generate electric fields via longitudinal and transverse piezoelectric effects. Figure 1c,d illustrate the process of transforming voltage. A sinusoidal AC voltage $V_{\mathrm{in}}$ is applied to the input, and due to the inverse piezoelectric effect, longitudinal and transverse vibrations are excited predominantly on the LN when the frequency reaches the resonant frequency. Due to the positive piezoelectric effect, the longitudinal vibrations generate an electric field parallel to the plate at the output of the LN and the transverse vibrations generate an electric field perpendicular to the plate at the output of the LN. In the electrode-free region between the input and output electrodes, where the electric flux does not leak into the air due to the high dielectric constant of the plate, the electric field $E_{L}$ along the plate generated by the longitudinal effect should dominate, and in the output electrode region, $E_{L}$ tends to short-circuit, and therefore, $E_{T}$ dominates [26]. Eventually, a higher output voltage is generated, mainly at the output electrode.

According to Nakamura et al. [26], using the side departing from the direction of polarization as the output electrode gives a higher output voltage. The step-up ratio reaches the highest value when the driving frequency is close to the resonant frequency of the LNT structure [15]. The voltage gain from the LNT is related to its geometry and piezoelectric properties, including the length of the piezoelectric *L*, the thickness of the piezoelectric *H*, the loss factor of the piezoelectric *Q*, the coupling factor for tensile vibration $k_{23}$, and the coupling factor for longitudinal vibration $k_{33}$. The product of $k_{23}$ and $k_{33}$ varies with the orientation of the LiNbO_3_ crystal and the step-up ratio of the transformer is proportional to $k_{23}k_{33}$. Thus, the 128° Y-cut LiNbO_3_ crystals in which the product of $k_{23}$ and $k_{33}$ is near a maximum value are usually chosen as the materials to manufacture transformers.

**Figure 2:**
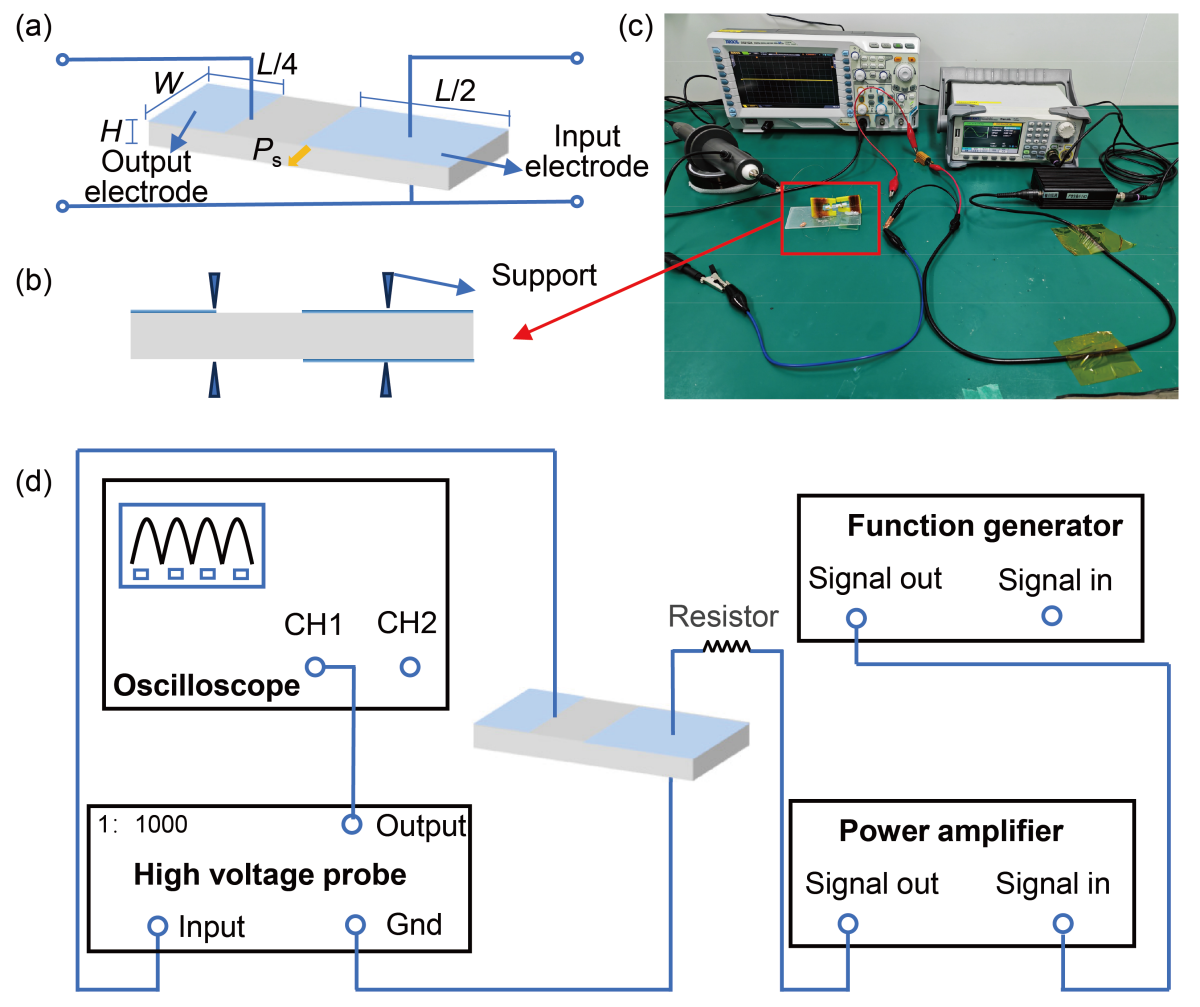
Schematic diagram of the LNT experiments: (**a**) Schematic diagram of LN dimensions. (**b**) Schematic of the LN fixture. (**c**) Physical connection diagram of experiments. (**d**) Circuit diagram of experiments.

By designing suitable configurations and circuits, it is possible to generate a voltage high enough to ionize air molecules at a low input voltage. In this paper, an LNT was first fabricated, and experiments with low input voltage–high output voltage were conducted. The feasibility of LNT to ionize air was verified. In this case, an LNT using a 29.1 (Length) × 6.3 (Width) × 1 (Height) mm, 128° Y-cut piece of LN, as shown in Figure 2a, was built and tested. The main structure of the LNT was fabricated, first, by laser cutting the PT, and then, by ion sputtering vanadium electrodes onto the input and output sections. The choice of electrode position affects the step-up ratio, while the polarization direction is the same as in Figure 2a. The input electrode is deposited on the right half of the top surface, covering a length of *L*/2. Similarly, the common ground electrode is deposited on the right half of the bottom surface, with the same length as the input electrode. The output electrode is deposited at the left end of the top surface, with a length of *L*/4.

Since the PT is driven at resonance frequency, its operation is closely dependent on its position. The resonance of the crystal can lead to breakage and even *Q*-factor degradation, making the effect of mechanical vibration on the crystal significant. Therefore, it is essential to fix the vibration nodes of the PT. Typically, the node positions experience minimal vibration but maximum stress. To maximize energy capture and gain, the energy is most efficiently collected at the nodes. In our model, two nodes were selected, located at *L*/4 from the two ends of the plates, as shown in Figure 2b. The PTs were secured using 3D-printed fixtures, as shown in Figure 2c, with copper wires (0.1 mm in diameter) connected to the input and output electrodes at the nodes using conductive silver adhesive.

Figure 2c,d shows the circuit connection where the signal generator was connected to the input electrode through a power amplifier with a resistor. Then, the output was connected to an oscilloscope via a high-voltage probe to detect voltage changes. Sinusoidal voltage with different frequencies and amplitudes was applied to the vertical crystal by a waveform generator.

**Figure 3:**
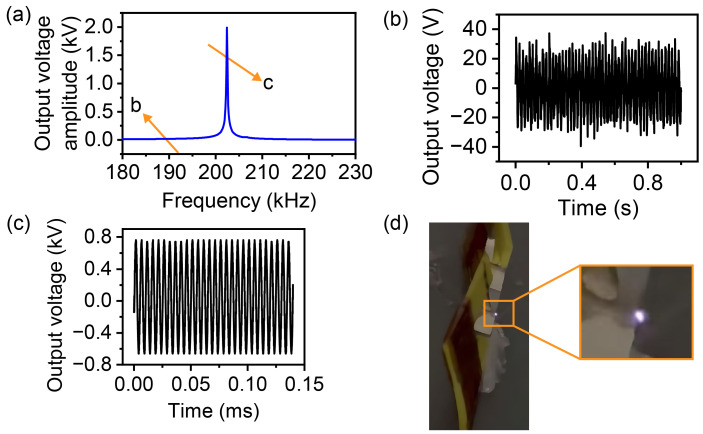
Schematic diagram of the frequency sweeping process: (**a**) Illustration of output voltage variation with frequency. (**b**) Spurious signals when there is no resonance. (**c**) Output voltage signal when the resonant frequency is reached. (**d**) Discharge caused by the high output voltage of the LN transformer driven at 8 Vpp, 201 kHz (see Supplementary Video S1).

In the experiments, we first estimate the approximate range of the second resonance frequency. Then, we sweep the frequency with a peak-to-peak value of 500 mV. A schematic diagram representing the variation of output voltage with input frequency is shown in Figure 3a. When there is no resonance, oscilloscope only captures spurious waves, as shown in Figure 3b. When resonance occurs, a significant jump in the output voltage is observed near the resonance frequency. The oscilloscope captures a sudden change in the signal. A high-voltage signal with a period consistent with the input frequency can be seen, as shown in Figure 3c. According to this approach, we change the input voltage, slightly adjust the input frequency, and then, the output voltage signal at the highest transformation ratio is captured. When the peak-to-peak value of the output voltage exceeds 1 kV, we can see whether the discharge phenomenon occurs. The discharge phenomenon is illustrated in Figure 3d.

### 2.2. Experimental Results

Electromechanical coupling efficiency is maximized at the resonant frequency. The experimental results show the output voltage amplitude increases significantly when the signal source frequency reaches the second resonant frequency. Frequencies below or above this point result in a significant drop in voltage amplitude.

Although the boost effect near the resonance is significant, the desired performance is not achieved without considering the influence of the external circuit. Further analysis reveals the energy conversion efficiency of piezoelectric materials is not only related to the frequency but also the structure of the external circuit. Based on this, we improved the circuit design by adding an 80 Ω resistor at the input, resulting in a higher step-up ratio.

When the output voltage difference is in the order of kilovolts for gas discharge, gas discharge would occur. After the improvement of the circuit, the step-up ratio increased. When the input peak-to-peak voltage was 8 V, the output voltage reached 2 kV, and the edge part of the output terminal discharged successfully. As shown in Figure 3d, a purple bright spot appeared near the edge of the output. The resonance frequency was 201 kHz.

## 3. Analysis of the Lithium Niobate Transformer

### 3.1. Simulation

To theoretically analyze the impact of the structure parameters on the performance of LNTs, simulations were conducted. In this study, we used COMSOL Multiphysics 6.2 (a multiphysics finite element analysis software), to perform the finite element analysis of the LN transformer.

We performed two types of simulations for LN as follows: one for the intrinsic frequency calculations and another for the piezoelectric properties. Firstly, the second-order longitudinal vibration frequency of LN was determined by calculating the intrinsic frequency and vibration characteristics of the LN plate. Then, the electromechanical coupling effects of the LN transformer were modeled by considering the interaction between the mechanical and the electrical field, solving for the distribution of both the electric field potential and the stress.

A three-dimensional finite element model of the LN plate was established for the simulation. The model was the same size as the sample described in Section 2, which was 29.1 mm in length, 6.3 mm in width, and 1 mm in thickness. LN is an anisotropic material, and its crystal axis direction is consistent with the *Z*-direction. The Y-cut was described by rotating the orientation of the plate in the global coordinate system. The material parameters of LN referred to Comsol 6.2 software [34]. The elasticity matrix parameters, coupling matrix parameters, and relative permittivity are listed in Table 1, Table 2 and Table 3. Other components not listed in the above tables are all zeroes. The density of LN is 4700 kg/m^3^ [34].

**Figure 4:**
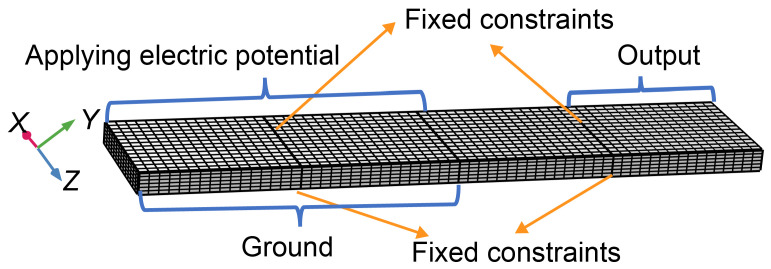
Schematic diagram of the boundary conditions and the mesh for simulation.

For the intrinsic frequency analysis, fixed constraints were applied on the upper and lower surfaces of the structure at the 1/4 and 3/4 positions, respectively. While performing the frequency domain analysis of piezoelectric characteristics, in addition to the fixed constraints imposed, a potential of 5 V was applied on the input side, and the output side was used as a circuit terminal for the analysis of output voltage. The schematic diagram of the boundary conditions and the mesh for simulation is illustrated in Figure 4.

**Figure 5:**
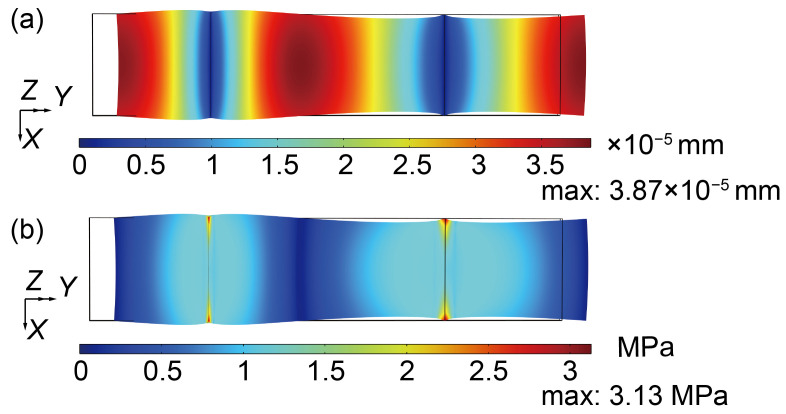
Contour of the displacement and stress of second-order longitudinal resonant frequency (192.16 kHz): (**a**) Contour of the displacement. (**b**) Contour of the stress.

Initially, the LN eigenfrequency was obtained. The displacement and stress results of second-order longitudinal intrinsic frequency (192.16 kHz) are presented in Figure 5. Figure 5a illustrates the displacement distribution across different parts of the transformer, with colors representing variations in displacement magnitude. As can be seen from the deformation shown in Figure 5, the main deformation of the formation at 192.16 kHz is along the length of the plate. Figure 5b shows the stress distribution, with the maximum stress occurring at the displacement constraints.

**Figure 6:**
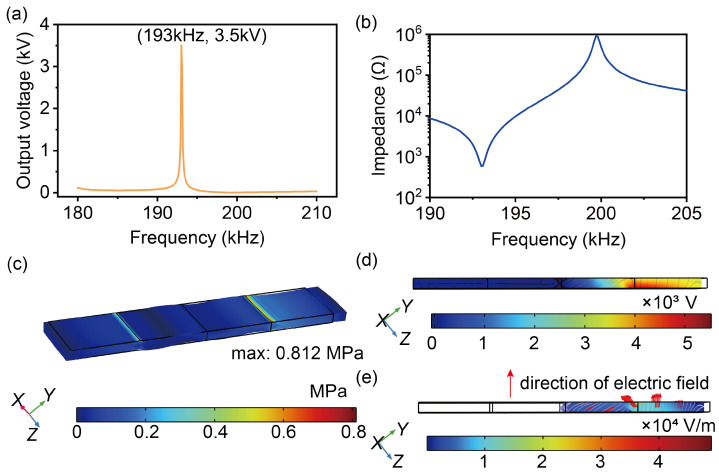
Curve of the output voltage and impedance versus frequency, as well as the contour of the stress, electric potential, and electric field: (**a**) Output voltage versus the frequency. (**b**) Impedance of LNT versus the frequency. (**c**) Contour of the stress at 193.0 kHz. (**d**) Contour of the electric potential at 193.0 kHz. (**e**) Contour of the electric field at 193.0 kHz.

Subsequently, the piezoelectric properties of the transformer were analyzed. Based on the frequency calculation results, the frequency sweep was performed near the second-order longitudinal intrinsic frequency. There is a significant increase in output voltage at 193.0 kHz, as shown in Figure 6a. The curve of the impedance of LNT versus the frequency was calculated, as shown in Figure 6b. During simulation, the mechanical loss factor was set to 0.0018 [27]. The input impedance showed a decrease near the resonant frequency of 193 kHz. When sweeping from low to high frequency, a low impedance was observed near the resonance, followed by a high-impedance region indicating anti-resonance. Figure 6c shows the stress distribution and formation at 193.0 kHz, which was similar to the results of the intrinsic frequency analysis. The maximum stress occurs at two nodal locations. Figure 6d illustrates the transformer’s electric potential distribution at 193.0 kHz. The maximum value is at the output. Figure 6e shows the electric field distribution at 193.0 Hz, where the electric field is maximum at the output node, in which the output voltage produces an electric field perpendicular to the plate, while the electrode-free region between the input and the output produces an electric field parallel to the plate. This is consistent with the conclusions in the literature [26].

### 3.2. Parametric Analysis

#### 3.2.1. Analysis of the Structure

**Figure 7:**
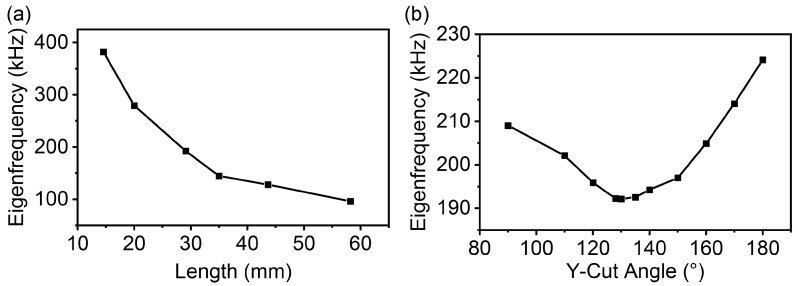
Eigenfrequency versus the length and the polarization direction of the LN: (**a**) Eigenfrequency versus the length of the LNT. (**b**) Eigenfrequency versus the polarization direction of the LNT.

In Section 3.1, we calculated the eigenfrequency of an LN crystal with COMSOL, and the results were in good agreement with the experimental data. To further investigate the effect of the size and polarization direction on the eigenfrequency of piezoelectric crystals, we varied the length, thickness, width, and polarization direction of the LN while keeping the other variables constant. The simulations showed that changes in thickness and width had a negligible effect on the eigenfrequency, while changes in length had a significant impact, as shown in Figure 7a. As the length decreases, the eigenfrequency increases, assuming the width and thickness are constant. The polarization direction also affects the eigenfrequency, as shown in Figure 7b. The polarization direction varied with the Y-cut angle θ, while the size of the LN was 29.1 (*L*) × 6.3 (*W*) × 1 (*H*) mm. The lowest second-order longitudinal intrinsic frequency was found when the Y-cut angle was close to 130°.

**Figure 8:**
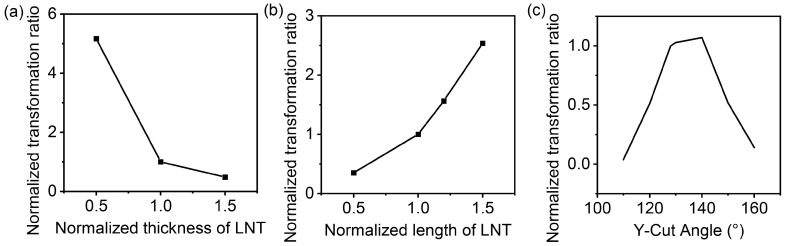
Normalized transformation ratio versus normalized structural parameters: (**a**) Normalized transformation ratio versus normalized thickness of LNT. (**b**) Normalized transformation ratio versus normalized length of LNT. (**c**) Normalized transformation ratio versus polarization direction of LNT.

The size of the LNT also has an effect on the step-up ratio. The effects of the above structural parameters on the step-up ratio were first analyzed by simulation. The results are plotted in Figure 8. The data were normalized by the dimensions of the experimental subjects in Section 2. As the LNT becomes longer and thinner, the step-up ratio of the transformer increases. The voltage gain is highest when the Y-cut angle is around 130°.

**Figure 9:**
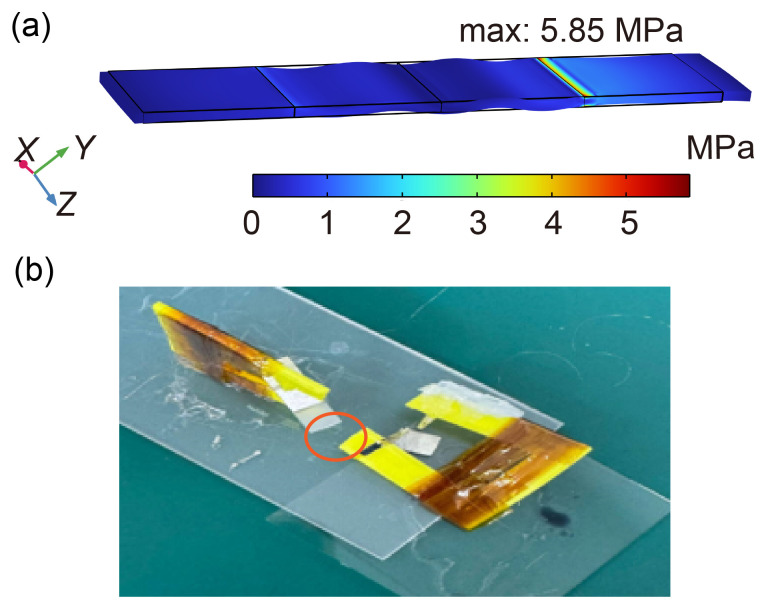
Analysis of the fracture of the 0.5 mm-thickn LNT: (**a**) Contour of the stress of the 0.5 mm-thick LNT at 208.1 kHz. (**b**) Picture of shattered LNT with a thickness of 0.5 mm after the experiment.

Considering that the LNT was designed to be used as a power unit on a microrobot, increasing the length of the LNT to increase the transformation ratio was not considered. Thinning the LN was further considered to obtain a higher transformation ratio, but it was found in the simulation calculations that thinning the LNT introduces a loss of strength. As shown in Figure 9a, the maximum stress at the resonance of the 0.5 mm-thick LNT is 5.85 MPa, which is much higher than that of the 1 mm-thick LNT. Experiments have similarly demonstrated this. The LNT had the same length and width as in Section 2, and the thickness of the LNT was 0.5 mm. The LNT had a second-order resonant frequency of about 202.8 kHz, which was close to that of the LNT with a thickness of 1 mm.

Although the step-up ratio increases as the thickness of the LNT decreases, the stress at the clamping location also increases and the transformer becomes fragile. During the experiment with the LN whose thickness was 0.5 mm, the boosting effect was effective near the resonance, but its strength was not sufficient, making it shatter during frequency sweeping, as shown in Figure 9b. The fracture occurred near the output of the LN while the input voltage was less than 1 Vpp. To address the issue of LNT fragility, we still used a crystal material with a thickness of 1 mm. When the output voltage was in the order of kilovolts, no lead breakage occurred, and the crystal was not fractured.

#### 3.2.2. Analysis of the Circuits

The LN is driven by resonance and typically operates in high-frequency mode, which requires more power than what an ordinary signal source can provide. In our experiment, due to the low power of the signal generator, the LNT operated inefficiently and could not function properly when driven solely by the signal transmitter. To solve this problem, a 20 W power amplifier was added at the input, successfully driving the transformer. A significant voltage boost was observed near the resonance frequency, as presented in Section 2. The 20 W power amplifier used in experiments is relatively bulky but can be significantly miniaturized to the centimeter scale for integration into centimeter-sized microrobots. For sub-centimeter-scale applications, further miniaturization of the external circuitry, including the power amplifier, would be necessary.

By adjusting the resistance of the input, it was found that the boost gain changed when different resistors were connected. During the experiment described in Section 2, an LNT was connected to an external resistor of 80 Ω. The set-up ratio achieved a voltage gain of over 250, produced an output voltage exceeding 2 kV, and successfully generated a discharge at an input voltage of only 8 Vpp. However, when the resistance was replaced by other resistance values, the boost effect was not as good as that of 80 Ω.

**Figure 10:**
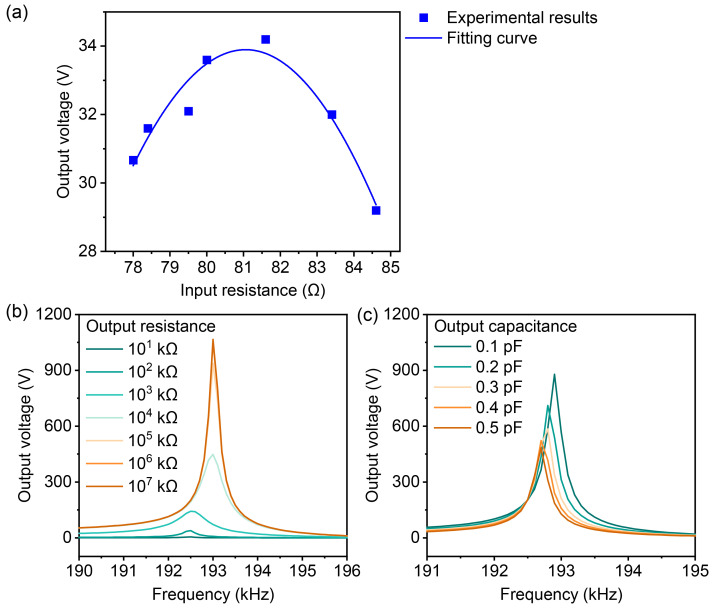
Effects of input and output circuit parameters on output voltage: (**a**) Output voltage versus the resistance in the input circuit when the input voltage was 2 Vpp. (**b**) Output voltage versus the frequency at different output resistances. (**c**) Output voltage versus the frequency at different output capacitance.

In order to further quantify the effect of the input circuit, experiments were conducted to observe the output voltage by varying the resistance in the input circuit. The results are plotted in Figure 10a. When the input voltage was 2 Vpp, the output voltage varied with the external resistance at the input. The experimental results indicate that as the resistance changes, the output voltage does not vary monotonically, but follows a parabolic curve. In Figure 10a, the output voltage reached its maximum near an external resistance of 82 Ω.

The performance and output voltage of the LNT can be improved through impedance matching in the input circuit, while the output design also influences the device’s performance. The impact of load resistance and load capacitance on the resonant frequency and voltage gain of the LNT was investigated through simulation, as shown in Figure 10b,c. As the resistance increases, the output voltage increases and approaches the open-circuit condition, while the resonant frequency also increases. On the other hand, as the capacitance increases, the output voltage and the resonant frequency decrease.

## 4. Design of the Discharge Thruster

The LNT is compact and lightweight, enabling the miniaturization of high-voltage power supplies. This advancement is expected to move from experimental research to practical applications, particularly in fields requiring miniaturized equipment and stringent environmental standards. Ion wind presents several advantages, including low noise levels, efficient power consumption, rapid response times, and the lack of mechanical moving components. However, using ion wind to power microrobots requires a miniature high-voltage source to excite discharge. Applying the LNT could solve this difficulty.

**Figure 11:**
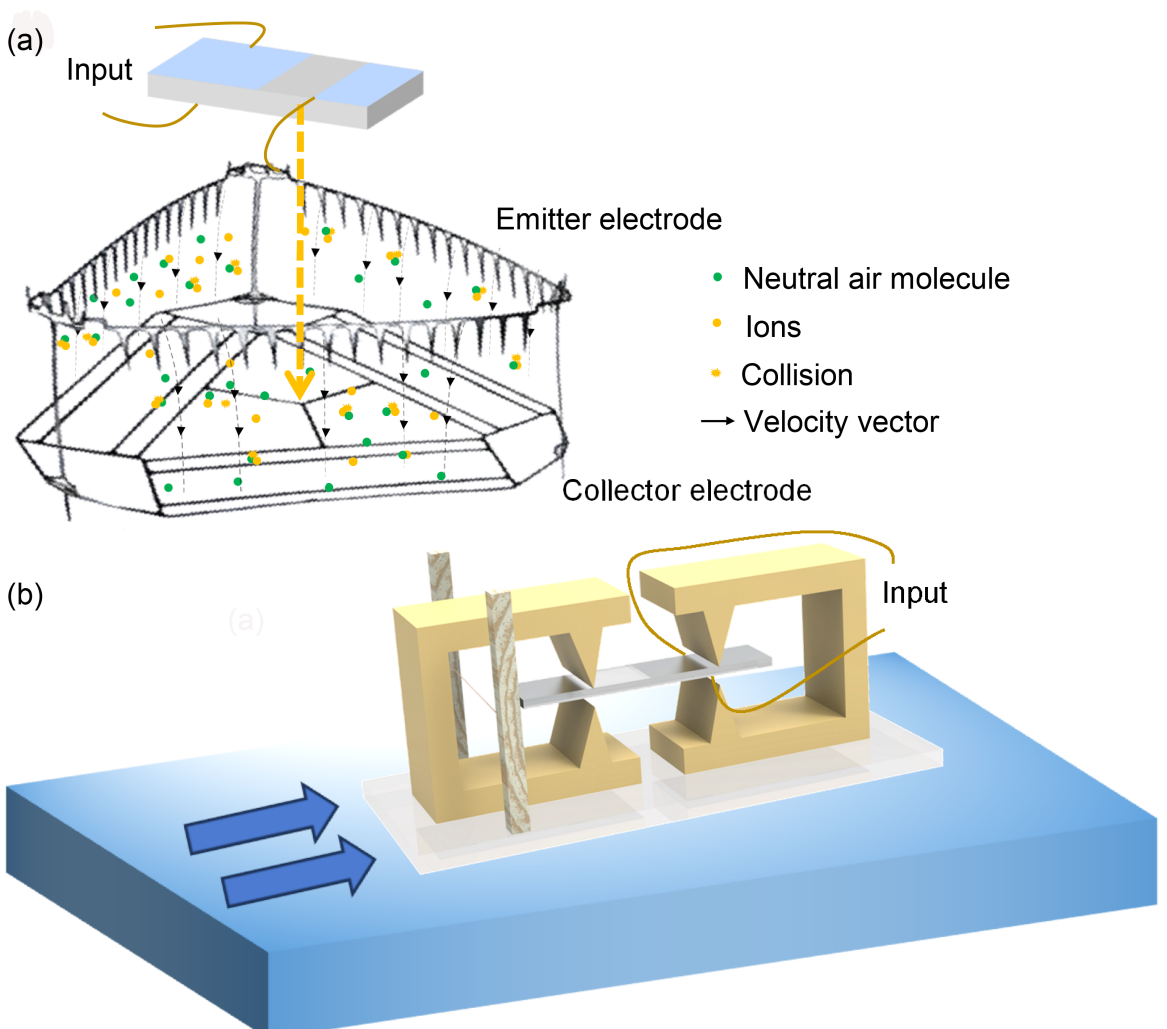
Application of the LNT: (**a**) Illustration of the LNT serving as a power source in a flying robot. (**b**) Illustration of the LNT generating ion wind for propulsion in a sailing robot.

We propose two design concepts using lithium niobate as a power source for microrobots. In our experiments, employing a proper structural design and circuit design, a voltage transformation ratio exceeding 200 with the lithium niobate transformer was achieved and ion wind generation was observed when the output voltage exceeded 2 kV. One concept utilizes the high voltage output to power the driving electrodes directly, while the other takes advantage of the ionizing discharge at the transformer’s output to generate ion wind for propulsion. The conceptual design of the LNT highlights its promising application potential, as shown in Figure 11.

As shown in Figure 11a, the output terminals of the lithium niobate transformer are connected to the ion wind emitter, providing high voltage to the emitter. Based on previous studies [7], by applying a high-voltage electric field near the emitter electrode, the gas near the emitter is ionized to form positive and negative ions. The ions accelerate under the influence of the electric field, resulting in an effect similar to avalanche breakdown. The ions continuously collide with gas molecules, transferring momentum and ultimately forming a macroscopic ion wind between the emitter and collector electrode. The resulting ionized wind successfully enabled the flying microbots to lift off. However, the high voltage in the previous study was provided by an external device. By carrying a LNT, a self-powered ion wind vehicle may be realized. The high voltage transformation ratio allows for the use of a low-voltage power supply to achieve the voltage threshold required for the ion wind vehicle to take off (approximately 4 kV) [7].

Figure 11b shows another application example, which uses a micro ship robot. The discharge phenomenon of LNTs at high voltage outputs can be used to develop a discharge thruster system. In this system, the output of a LNT is used as the emitter electrode and a copper wire is set slightly farther from the end of the clamping device as the collector electrode, as shown in Figure 11b. When the LNT outputs a high voltage, the air in the vicinity of the emitter is ionized, similar to the process of ionization in aircraft, creating ion wind. Combined with a buoyant structure, the thruster is able to be placed on the surface of the water, thus enabling the microrobot to move on the water surface. In this way, the LNT not only demonstrates ionization properties at high voltage outputs but also provides an innovative propulsion solution for the movement of microrobots on water.

## 5. Conclusions

In summary, a compact LN single-crystal transformer was designed, fabricated, and analyzed. The experimental results indicate that frequency and the external circuit are critical factors in achieving a high step-up ratio. COMSOL simulations demonstrate that the intrinsic frequency of the transformer is dependent on its length, and the voltage amplification effect peaks near the resonant frequency. Notably, the simulation results align well with the experimental findings. The experiments further reveal that the 128° Y-cut LNT can generate voltages of several thousand volts, successfully inducing corona discharge, thereby enabling the generation of ion wind. In the optimization of the system, frequency tuning and external circuit design are pivotal for achieving efficient energy conversion. Future work could focus on further optimizing the circuit design, enhancing system stability, and ensuring consistent discharge performance. Additionally, efforts could be directed toward the development of a miniaturized power supply integrating the LNT, enabling the generation of ion wind to drive the motion of microbots.

## Figures and Tables

**Table 1:** Elasticity matrix parameters of LN [34].

**Component**	$c_{11}$	$c_{12}$	$c_{13}$	$c_{14}$	$c_{22}$	$c_{23}$
Value (GPa)	202.90	52.92	74.91	9.00	202.90	74.91
**Component**	$c_{24}$	$c_{33}$	$c_{44}$	$c_{55}$	$c_{56}$	$c_{66}$
Value (GPa)	−9.00	243.08	59.90	59.90	8.99	74.88

**Table 2:** Coupling matrix parameters of LN [34].

**Component**	$e_{21}$	$e_{31}$	$e_{22}$	$e_{32}$
Value (C/m^2^)	−2.54	0.19	2.54	0.19
**Component**	$e_{33}$	$e_{24}$	$e_{15}$	$e_{16}$
Value (C/m^2^)	1.31	3.70	3.70	−2.53

**Table 3:** Relative permittivity of LN [34].

Component	$\varepsilon_{11}$	$\varepsilon_{22}$	$\varepsilon_{33}$
Value	43.60	43.60	29.16

## Data Availability

Data in the paper are available from the authors.

## Supplementary Materials

The following supporting information can be downloaded at: https://www.mdpi.com/article/10.3390/mi16030277/s1, Video S1: The discharge at the output edge of LNT driven by 8 Vpp, 201 kHz sine wave.

## Abbreviations

The following abbreviations are used in this manuscript:

AC	Alternating current
DBD	Dielectric barrier discharge
DC	Direct current
LN	Lithium niobate
LNT	Lithium niobate transformer
PT	Piezoelectric transformer
